# Risk of low levels of blood group antibodies mediating hemolysis in ABO-incompatible neonates with negative three hemolysis tests

**DOI:** 10.3389/fped.2024.1392308

**Published:** 2024-08-05

**Authors:** Hongxing Lin, Pingxiang Luo, Chen Liu, Xiaosong Lin, Chengwen Que, Wenhui Zhong

**Affiliations:** ^1^ Department of Blood Transfusion, Fujian Maternity and Child Health Hospital, Fuzhou, Fujian, China; ^2^ Department of Neonatology, Fujian Maternity and Child Health Hospital, Fuzhou, Fujian, China; ^3^ Clinical Laboratory, Fujian Maternity and Child Health Hospital, Fuzhou, Fujian, China

**Keywords:** ABO hemolytic disease of the newborn, blood group antibodies, three hemolysis tests, mean corpuscular hemoglobin concentration, coefficient of variation of red blood cell volume distribution width

## Abstract

**Objective:**

To explore the risk of low-level blood group antibody-mediated hemolysis in ABO-incompatible newborns with negative three hemolysis tests, aiming to assist in the identification and management of neonatal jaundice.

**Methods:**

A retrospective case-control study was performed in 892 children with jaundice. The patients were divided into three groups: group I, ABO compatible, negative three hemolysis tests; group II, ABO incompatible, negative three hemolysis tests; and group III, ABO incompatible, positive three hemolysis tests. We analyzed the differences in clinical data, blood routine and biochemical laboratory results.

**Results:**

(1) Patients in group II had higher levels of mean corpuscular volume (MCV), standard deviation of red blood cell volume distribution width (RDW-SD), alanine aminotransferase (ALT), lactate dehydrogenase (LDH), alkaline phosphatase (ALP), and bile acid (BA) than those in group I (*P* < 0.05). However, there were no statistically significant differences in the MCV, ALT, ALP and BA levels between groups II and III (*P* > 0.05). (2) Mean corpuscular hemoglobin concentration (MCHC) >359.5 g/L, cell volume distribution width (RDW-CV) >15.95%, and reticulocyte count (RET) >4.235% were identified as independent predictors of positive hemolysis test results (*P* < 0.001). The combination of MCHC, RDW-CV, and RET% yielded an AUC of 0.841.

**Conclusion:**

Low-level blood group antibody-mediated hemolysis may occur in ABO-incompatible neonates even when three hemolysis tests are negative. Changes in liver function parameters must be monitored. The combination of MCHC, RDW-CV, and RET% can be used to improve the detection rate of HDN.

## Introduction

Hemolytic disease of the newborn (HDN) is an immune hemolytic disease caused by differences in blood group antigens of the red blood cells of a mother and infant. During pregnancy, the maternal immune system is stimulated by fetal allogeneic red blood cell antigens to produce immunoglobulin G antibodies (1) that cross the placental barrier and destroy fetal red blood cells, resulting in fetal or newborn hemolytic anemia, jaundice, edema, and hyperbilirubinemia (2). The diagnostic criteria for ABO-HDN include ABO incompatibility between the mother and newborn, clinical manifestations of neonatal bilirubin abnormalities, and serological confirmation of the presence of hemolytic antibodies using three hemolysis tests (3, 4). However, the three hemolysis tests are qualitative, and false-negative results may occur (5). It is not yet possible to identify which ABO-incompatible neonates with negative hemolysis test results have undetected low levels of blood group antibodies or determine if these low levels of antibodies cause severe hemolysis. Therefore, this study reviewed the clinical data and laboratory indicators related to ABO-HDN and explored the possibility of low-level blood group antibodies mediating hemolysis in ABO-incompatible neonates with negative hemolysis test results, aiming to assist in the identification and management of neonatal jaundice.

## Materials and methods

### Research objectives

This study included 892 children with jaundice who were hospitalized in the Neonatology Department of Fujian Maternity and Child Health Hospital between June 2020 and May 2023. The children were full-term infants with a gestational age >37 weeks and a birth weight >2,500 g. Exclusion criteria for cases included (1) HDN caused by non-ABO blood group antibodies; (2) other causes of pathological jaundice, such as sepsis, G6PD deficiency, hemoglobinopathy, and cranial hematoma; (3) diseases of the liver and biliary system; and (4) other exclusion factors such as premature birth, low birth weight and incomplete clinical data. The data of 1,244 patients were collected, though 352 patients were excluded, resulting in the final analysis of the data of 892 patients.

All children underwent three hemolysis tests to investigate the cause of jaundice. Gender, gestational age, birth weight, mother's ABO blood type, length of hospitalization, age at the time of examination, and results of laboratory tests during hospitalization (including ABO blood type, three hemolysis tests, routine blood tests, and biochemical tests) were retrospectively collected and analyzed. The values of red cell indices were obtained to get the information regarding red cell destruction, such as red blood cell count (RBC), hemoglobin (Hb), mean corpuscular volume (MCV), mean corpuscular hemoglobin (MCH), mean corpuscular hemoglobin concentration (MCHC), coefficient of variation of red blood cell volume distribution width (RDW-CV), standard deviation of red blood cell volume distribution width (RDW-SD), reticulocyte count (RET), and reticulocyte percentage (RET%). Biochemical markers related to jaundice and liver function included total serum bilirubin (TSB), indirect bilirubin (IBIL), TSB/albumin ratio (B/A), alanine aminotransferase (ALT), aspartate aminotransferase (AST), AST/ALT ratio, lactate dehydrogenase (LDH), alkaline phosphatase (ALP), γ-glutamyl transferase (GGT), and bile acid (BA). The data were part of the daily diagnosis and treatment of the children during hospitalization, and the datasets were obtained from the hospital's information system.

### Diagnostic criteria

(1) The diagnostic criteria for ABO-HDN were ABO blood type incompatibility between mother and child, and among the results of three hemolysis tests, including direct anti-human ball test (DAT) and RBC antibody identification (containing free antibody test and antibody release test), any two positive tests or a single positive release test (6).
(2) The diagnostic criterion for anemia was Hb < 145 g/L (7).
(3) The diagnostic criterion for hyperbilirubinemia was TSB ≥ 256 umol/L in term infants (8).

### Methods

#### Grouping method

The patients were divided into three groups based on the maternal and infant ABO blood type compatibility and the serological diagnosis of HDN: group I, ABO compatible, negative three hemolysis tests; group II, ABO incompatible, negative three hemolysis tests; and group III, ABO incompatible, positive three hemolysis tests.

#### Detection method

Blood type identification, free antibody experiments, and antibody release experiments were performed using the microcolumn gel method, and the direct anti-human sphere test was performed using the test tube method. Routine blood, biochemical, and other indicators were detected using fully-automated instrument analysis. The experimental sample requirements and testing operations were based on the “National Clinical Laboratory Procedures” (9).

#### Instruments and reagents

The blood type test card, anti-globulin card, and supporting WADiana automatic blood type tester were purchased from Beijing Banpers Technology and Trade Co. Ltd. Anti-globulin antibody reagents were purchased from Shanghai Blood Biomedicine Co., Ltd. A Sysmex XN-2000 automatic hematology analyzer was used for routine blood testing, and an Abbott CI16200 automatic biochemical analyzer was used for biochemical testing.

#### Statistical analysis

All statistical analyses were conducted using SPSS 22.0. Normally-distributed continuous data are expressed as mean and standard deviation while those with skewed distributions are expressed as median and interquartile range. The continuous data were compared using the rank sum test of multiple independent samples, and post-hoc multiple comparisons were made using the Kruskal-Wallis one-way analysis of variance. The chi-square test was used to compare categorical data. The correlation between each indicator and positive hemolysis test results were determined using correlation ratios. Receiver operating characteristic (ROC) curves were used to identify index and cutoff values for HDN screening. A binary logistic regression analysis was performed to test the significance of the cutoff values. Statistical significance was set at *P* < 0.05.

## Results

### Clinical and laboratory parameters

All results are presented in Table 1. The gestational age and birth weight differed significantly between groups II and III (*P* < 0.05). Given that all children in the three groups were full-term infants with birth weights exceeding 2,500 g, the differences in gestational age and birth weight were considered negligible. The frequency of ABO incompatibility was higher in female children than in male patients (59.8% vs. 41.3%, *P* < 0.05). However, the rate of positive hemolysis results did not differ between female and male patients with ABO incompatibility (63.3% vs. 56.8%, *P* > 0.05). There was a significant difference in the age at the time of examination among the three groups (*P* < 0.05), with the oldest in group II and the youngest in group III.

**Table 1:** Comparison of clinical and laboratory parameters.

Parameter	I (*n* = 264)	II (*n* = 250)	III (*n* = 378)	*P*
Gender				
Male (*n*)	182	128	168^b^	<0.05
Female (*n*)	82	122	210	
Gestational age (W)	39.18 ± 1.09	39.07 ± 1.10^a^	39.37 ± 1.05^c^	<0.05
Birth weight (g)	3,295.17 ± 375.6	3,233.14 ± 348.69^a^	3,329.70 ± 365.93^c^	<0.05
Length of hospitalization (days)	4 (3–6)	4 (3–5)^a^	5 (4–7)	<0.05
Age at the time of examination (days)	4 (1–7)	5 (3–7)	2 (1–4)	<0.05
RBC (10^12^/L)	4.83 (4.42–5.23)	4.80 (4.36–5.20)^a^	4.30 (3.77–4.78)	<0.05
Hb (g/L)	169 (152–181)	166 (152–183)^a^	154 (138–170)	<0.05
MCV (fL)	94.7 (91.7–97.8)	97.8 (94.7–101.1)	98.4 (95.4–101.7)^b^	<0.05
MCH (pg)	35.0 (34.0–35.8)	34.9 (34.0–36.2)^a^	36.0 (34.9–37.1)	<0.05
MCHC (g/L)	368 (361–375)	357 (349–367)	365 (359–371)	<0.05
RDW-CV (%)	15.3 (14.8–15.9)	15.3 (14.7–15.9)^a^	16.4 (15.5–17.4)	<0.05
RDW-SD (fL)	52.8 (50.6–55.9)	54.8 (51.6–58.4)	57.5 (54.0–61.9)	<0.05
RET (10^9^/L)	78.1 (46.3–140.5)	100.7 (56.0–181.9)	190.3 (106.3–248.5)	<0.05
RET% (%)	1.73 (0.97–3.12)	2.20 (1.19–3.82)	4.28 (2.40–5.96)	<0.05
TSB (μmol/L)	259.7 (160.2–308.3)	257.6 (164.5–311.4)	252.7 (196.2–303.2)	0.629
IBIL (μmol/L)	248.8 (169.5–296.3)	250.1 (181.7–300.6)	239.3 (184.8–285.9)	0.584
B/A ratio	7.45 (5.53–8.70)	7.31 (5.46–8.66)	7.25 (5.57–8.54)	0.462
ALT(U/L)	10.79 (7.63–14.58)	12.10 (9.09–16.15)	11.8 (8.9–15.7)^b^	<0.05
AST(U/L)	35.4 (27.8–47.4)	37.4 (28.8–48.1)^a^	42.9 (31.0–56.9)	<0.05
AST/ALT ratio	3.29 (2.47–4.91)	3.16 (2.29–4.06)^a^	3.61 (2.71–4.93)	<0.05
LDH(U/L)	407.5 (333.8–523.8)	437.1 (366.1–550.7)	505.7 (417.3–615.2)	<0.05
ALP(U/L)	157.8 (127.3–194.6)	176.7 (145.2–209.2)	170.0 (142.1–199.4)^b^	<0.05
GGT(U/L)	139.3 (94.6–193.6)	145.0 (97.7–214.4)	136.5 (91.5–194.2)	0.073
BA (μmol/L)	9.4 (6.0–15.0)	11.7 (7.9–17.9)	11.3 (6.8–17.6)^b^	<0.05

^a^ *P*, ^b^*p* and ^c^*p* > 0.05.

^a^ *P* represents comparisons between group II and group I.

^b^ *p* represents comparisons between group III and group II.

^c^ *p* represents comparisons between group III and group I.

The TSB, IBIL, and B/A levels did not show significant differences among the three groups (*P* > 0.05). The length of hospitalization, RBC, and Hb levels were similar between groups I and II (*P* > 0.05). However, the MCV, RDW-SD, ALT, LDH, ALP, and BA levels were increased in group II (*P* < 0.05). There were no statistically significant differences in the MCV, ALT, ALP and BA levels between groups II and III (*P* > 0.05).

The numbers of children with anemia and hyperbilirubinemia in group I were 41 and 140, respectively. In group II, the corresponding numbers were 41 and 135, respectively. The frequencies of anemia or hyperbilirubinemia were not significantly different between the patients in groups I and II (15.5% vs. 16.4% and 53.0% vs. 54.0%, respectively; *P* > 0.05).

### Correlations between indicators and positive hemolysis results

The RDW-SD, AST, AST/ALT ratio, and LDH showed weak correlations with positive hemolysis results (*P* < 0.001). Meanwhile, the RBC, Hb, MCH, MCHC, RDW-CV, RET, and RET% demonstrated moderate correlations with positive hemolysis test results (*P* < 0.001), as illustrated in Table 2.

**Table 2:** Correlations between indicators and positive hemolysis results.

Parameter	E^2	F	*P*
RBC	0.099	68.93	<0.001
Hb	0.06	40.23	<0.001
MCH	0.063	42.254	<0.001
MCHC	0.068	46.926	<0.001
RDW-CV	0.117	82.967	<0.001
RDW-SD	0.047	31.62	<0.001
RET	0.114	81.108	<0.001
RET%	0.132	96.307	<0.001
AST	0.02	12.845	<0.001
AST/ALT ratio	0.015	9.651	0.002
LDH	0.031	20.455	<0.001

### ROC curve analysis of indicators for HDN screening

**Figure 1:**
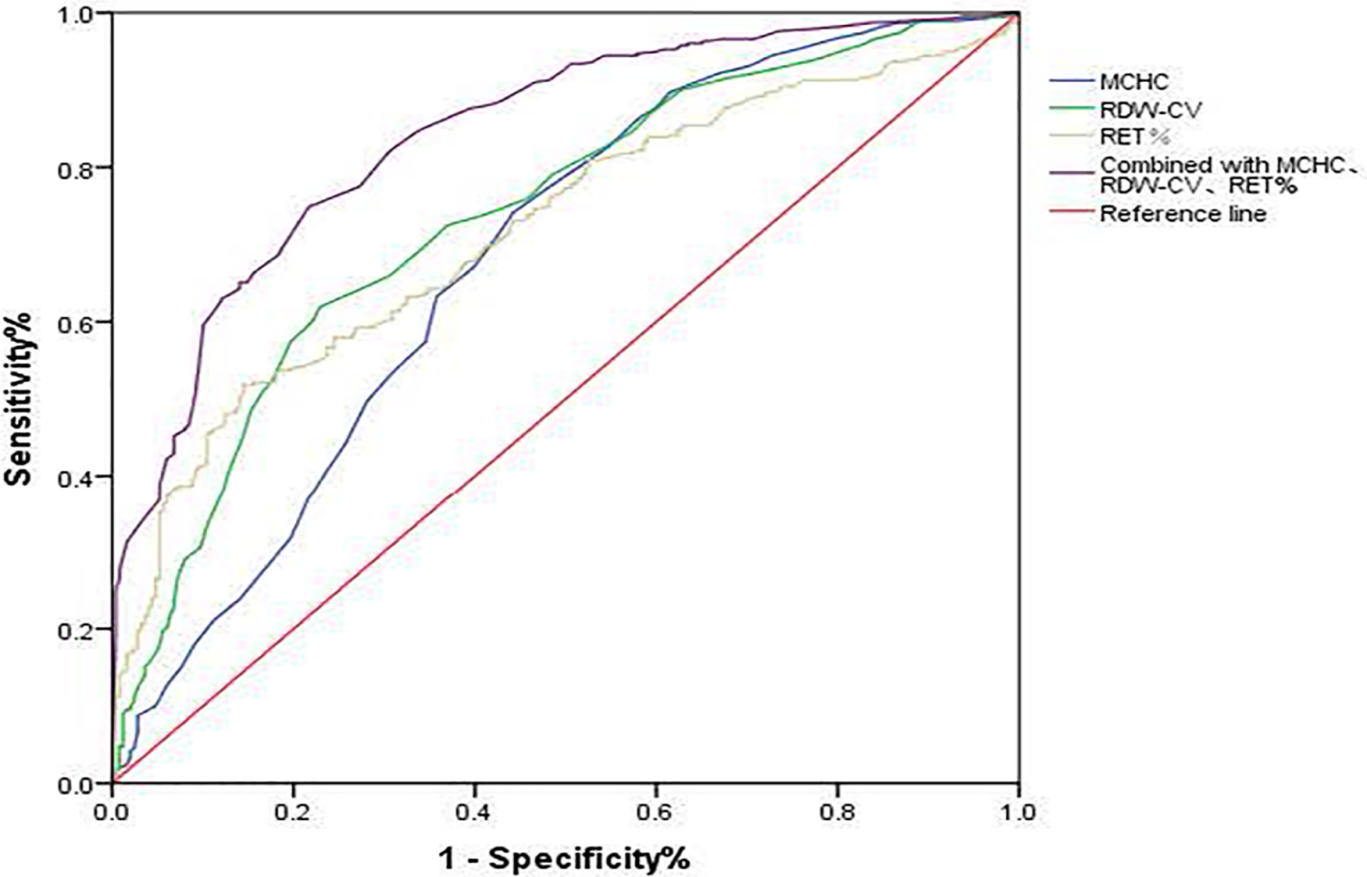
ROC curve of MCHC, RDW-CV, and RET% for HDN screening. MCHC, mean corpuscular hemoglobin concentration; RDW-CV, coefficient of variation of red blood cell volume distribution width; RET%, reticullocyte percentage.

In both ABO-incompatible groups, the RBC, Hb, MCH, MCHC, RDW-CV, RET, and RET% proved valuable for screening HDN, with each index having an AUC greater than 0.600 (*P* < 0.001). The cutoff values for HDN screening are presented in Table 3 and Figure 1.

**Table 3:** ROC curve analysis of indicators for HDN screening.

Parameter	AUC	Cutoff	Sensitivity%	Specificity%	*P*
RBC	0.695	4.47	69.6%	58.7%	<0.001
Hb	0.651	159.5	62.8%	59.5%	<0.001
MCH	0.664	35.25	69.0%	57.8%	<0.001
MCHC	0.683	359.5	73.9%	56.5%	<0.001
RDW-CV	0.736	15.95	61.9%	77.1%	<0.001
RET	0.699	186.5	51.6%	78.0%	<0.001
RET%	0.724	4.235	52.1%	85.9%	<0.001
Combined with MCHC, RDW-CV, RET%	0.841	–	74.9%	78.3%	<0.001

### Effects of MCHC, RDW-CV, and RET% on HDN detection rate

Following logistic regression analysis, MCHC > 359.5 g/L, RDW-CV > 15.95%, and RET% > 4.235% emerged as independent predictors of positive hemolysis test results (*P* < 0.001), as illustrated in Table 4. When combined, MCHC, RDW-CV, and RET% produced an AUC of 0.841, with both sensitivity and specificity surpassing those of each individual indicator (Table 3 and Figure 1).

**Table 4:** Effects of MCHC, RDW-CV, and RET% on HDN detection rate.

Parameter		Univariable	*P*	Multivariable	*P*_adjust_
		OR (95% CI)		OR_adjust_ (95% CI)	
RBC	≤4.47	1.816 (0.962–3.426)	0.066	–	–
	>4.47^R^				
Hb	≤159.5	1.799 (0.959–3.373)	0.067	–	–
	>159.5^R^				
MCH	≤35.25^R^	1.543 (0.999–2.358)	0.051	–	–
	>35.25				
MCHC	≤359.5^R^	8.056 (5.063–12.819)	<0.001	7.904 (4.949–12.623)	<0.001
	>359.5				
RDW-CV	≤15.95^R^	4.876 (3.039–7.822)	<0.001	3.786 (2.298–6.238)	<0.001
	>15.95				
RET	≤198.75^R^	1.389 (0.685–2.820)	0.362	–	–
	>198.75				
RET%	≤4.235^R^	5.203 (3.126–8.659)	<0.001	3.769 (2.193–6.477)	0.001
	>4.235				

OR values were adjusted for age of Three Hemolysis Tests; R, references group.

## Discussion

HDN is an immune hemolytic disease, and it is the primary risk factor for neonatal pathological jaundice (10). ABO incompatibility is the main cause of hemolytic hyperbilirubinemia, and Clinical symptoms of jaundice appear between two and five days after birth (11). In China, prenatal examinations do not routinely screen for ABO-HDN-related antibodies. The laboratory diagnosis of ABO-HDN is typically based on three hemolysis tests performed after birth. False negative results are sometimes obtained due to weak erythrocyte antigens in newborns, untimely sample detection, the consumption of antibodies for blood type substances in the serum, limitations of the qualitative tests, and differences in result determination. Approximately 20% of newborns with ABO blood group incompatibility are diagnosed with HDN (12).

In this study, the bilirubin levels and B/A ratio, which was used to indicate the risk of hyperbilirubinemia, were not different between the groups, which is consistent with previously reported results (13, 14). This may be due to the fact that the patients included in this study had clinical symptoms of jaundice and that the bilirubin level cannot be used to determine the cause of jaundice. Most patients in group II underwent laboratory testing at approximately five days of age, which was later than that in the other groups. It was observed that jaundice progressed slowly in group II. Therefore, the late monitoring of jaundice should not be overlooked in ABO-incompatible patients. In addition, there was no statistical difference in the length of hospitalization between groups I and II. The patients in the two groups had similar RBC, Hb levels, and frequencies of anemia and hyperbilirubinemia. Therefore, we believe that even if children in group II have a low level of blood group antibodies, it is unlikely to cause severe hemolysis. Moreover, the progression of hemolysis is slow and does not lead to significant abnormalities in bilirubin and hemoglobin levels.

As HDN may cause diffuse liver cell damage, laboratory indicators reflecting liver function were also analyzed in this study. Patients in group II had higher levels of ALT, LDH, ALP, and BA than those in group I. However, increased enzyme levels typically indicate liver injury and a reduced capacity of the liver to process bilirubin (15–18)^.^ These findings suggest that the liver function differed between the two groups due to varying degrees of liver damage. The ALT, ALP, and BA levels did not differ between group II and group III, suggesting comparable liver function. Therefore, it is worth noting that even though patients in group II did not have severe hemolysis, their liver function and ability to metabolize bilirubin decreased.

The diagnosis of HDN includes serological tests to confirm the presence of hemolytic antibodies; however, low levels of antibodies may be missed, making it impossible to identify all patients with immune hemolysis. Immune hemolysis leads to the destruction and dissolution of red blood cells, which can cause abnormal volume and morphology of peripheral blood red blood cells. MCV and RDW are objective parameters that reflect the volume and volume heterogeneity, respectively. In this study, the MCV and RDW-SD in group II were higher than those in group I, though the RDW-CV was not significantly different. Additionally, the MCHC, RET, and RET% were higher in group II than in group I, and these indicators were correlated with positive hemolysis test results. This correlation was stronger than that between AST or LDH and positive hemolysis test results. Abnormal AST and LDH levels and their correlation with positive hemolysis results have been reported (19, 20). These differences in erythrocyte compensatory changes after hemolysis suggests that the causes of hemolysis differ between the two groups of patients with negative hemolysis test. In addition, the RDW-CV and RDW-SD in group II were lower than in group III, while the MCV was similar between the groups, suggesting that the destruction and dissolution processes of red blood cells are comparable between group II and group III, but the intensity of hemolysis in group II was slightly weaker. Therefore, clinicians should be aware of hemolysis due to low levels of blood group antibodies in children in group II.

In this study, MCHC > 359.5 g/L, RDW-CV > 15.95%, and RET% > 4.235% were independent predictors of positive hemolysis test results. When maternal and infant ABO blood types are incompatible, these indicators are helpful in identifying patients who should undergo screening for HDN. The AUC, sensitivity, and specificity of the combination of these three indicators were greater of those of each individual indicator. Therefore, these indicators can be used to increase the detection rate of ABO-HDN, thereby helping determine the cause of jaundice in children with ABO incompatibility and improving the management of neonatal jaundice.

In reality, sometimes the same child's hemolysis test result is negative before admission, and reexamination after admission has a weakly positive result. During hospitalization, some patients experienced recurrent jaundice, but on repeat testing, the hemolysis test results were still negative. Female patients had a higher probability of ABO incompatibility in this study, though the ABO-HDN positivity rate did not differ between male and female patients. These results suggest that low-level blood group antibody-mediated hemolysis must be considered, especially in female patients.

Our study had certain limitations. Patients with multiple causes of jaundice were excluded in this study. The interaction between HDN and other causes lacked sufficient observation. Therefore, it is necessary to continue to expand the data to include cases with multiple jaundice etiologies for comparison and to raise further awareness to the application value of combining two of the three indicators (MCHC > 359.5 g/L, RDW-CV > 15.95%, and RET% > 4.235%). HDN-negative children with recurrent jaundice, especially those sent again for hemolysis three tests, will be in the focus of our follow-up observation. In addition, the indicators used in this study may change only after the immune hemolysis of red blood cells reaches a certain level, as they are indirect indicators and lag behind the development of ABO-HDN. Therefore, for children with ABO-incompatible hyperbilirubinemia, especially those with negative hemolysis test results and changes in liver function, more sensitive methods (such as flow cytometry) should be considered to qualitatively or quantitatively detect blood group antibodies.

## Conclusions

In summary, ABO-incompatible newborns with negative three hemolysis tests may have low-level blood group antibody-mediated hemolysis. Red blood cells may undergo heterogeneous changes caused by immune hemolysis that is not severe. However, the changes in liver function caused by hemolysis must be monitored. MCHC > 359.5 g/L, RDW-CV > 15.95%, and RET% > 4.235% were identified as independent predictors of positive hemolysis test results. The combination of these predictors can help improve the detection of ABO-HDN and facilitate the management of jaundice.

## Data Availability

The raw data supporting the conclusions of this article will be made available by the authors, without undue reservation.
